# Macroglossia

**Published:** 1903-11-28

**Authors:** 


### MACROGLOSSIA.

Mr. M. R. Jay[1] records an interesting case of this affection which was apparently cured by the administration of thyroid extract. The child was five months old, stunted in growth, with a vacant look. The tongue was greatly enlarged, and hung down out of the mouth as low as the chin; it was purplish in colour, the upper surface was dry and fissured. The abdomen was greatly distended, the superficial veins were enlarged, and there was an umbilical hernia. Prolapse of the rectum was also present. The skin of the limbs and body was markedly dry.

On an attempt being made to examine the back of the tongue and throat the child ceased breathing, became deeply cyanosed, while convulsive twitchings of the limbs ensued. This was followed by gasping breaths, and a slow return to consciousness, the lividity not completely disappearing for an hour or two later. Similar attacks were of frequent occurrence—one every 24 hours, or oftener. Pulling out the tongue, lifting forward the lower jaw, and altering the child's position seemed to make no difference in the occurrence of the attacks.

There was an uncertain history of a chancre in the father's case nine years previously which was not followed by secondaries.

The child was treated medically for the next four months, mercury inunction and bromides internally being employed. Operation was not thought to be suitable because the base of the tongue was affected equally with the anterior part. However, the child did not improve, and at nine months he resembled a baby of three months old. His apathy, the dryness of his skin and other points led to a trial of thyroid gland treatment. Symptoms of improvement then rapidly showed themselves, and in less than two months the tongue had so diminished in size that it no longer protruded from the mouth. Swallowing and breathing had become practically normal, and no fit of any kind had taken place from a fortnight after commencement of the treatment. Rapid growth took place, and mental apathy disappeared. Mr. Jay concludes from this case that thyroid extract might possibly be used with advantage not only in other cases of macroglossia but also in cases of enlargement of the tonsils, with post-nasal growths and the frequent accompanying symptoms of arrested physical and mental development.

[1] Austral. Med. Gaz., Aug. 20.

